# Hate speech and hate-based harassment in online games

**DOI:** 10.3389/fpsyg.2024.1422422

**Published:** 2025-02-19

**Authors:** Garrison Wells, Ágnes Romhányi, Constance Steinkuehler

**Affiliations:** Department of Informatics, Donald Bren School of Information and Computer Sciences, University of California, Irvine, Irvine, CA, United States

**Keywords:** games, extremism, toxicity, survey, hate speech

## Abstract

The proliferation of hate speech and hate-based harassment has become a worryingly common trend in online gaming spaces, with researchers fearing that it could lead to the normalization of hateful behaviors on such platforms. However, little research has been done assessing the frequency of such events and how players respond to their occurrence. In this study, we conduct a large-scale survey (*n* = 602) asking players to reflect on their experiences and responses to hateful conduct in online games. We examine their perspectives when faced with hate speech and harassment from the role of a bystander, a victim, or the perpetrator. We then compare these responses with various demographic factors and personality traits to determine which variables might predict such conduct to occur and persist over time. Our findings suggest that hate speech and harassment are more accepted by those who are not directly targeted, potentially leaving those players as the remaining few to continue inhabiting and shaping online gaming spaces over time.

## 1 Introduction

Over the last two decades, the United States has witnessed a dramatic rise in extremist groups and rhetoric in both political discourse and everyday communication, both in-person and online (Riccardi, 2023; Youngblood, 2020). Online game platforms are no exception. In their most recent report on hate and harassment in online games, the Anti-Defamation League (Anti-Defamation League, 2024) found that 15% of adults and 9% of youth (ages 13–17) were exposed to white supremacist ideologies in online game platforms, most commonly in the form of hate speech and hate-based harassment directed at marginalized players. Such events are becoming a regular occurrence, with 30% of teens and adults reporting being exposed to these dangerous ideologies at least once a week. Given the popularity of online games among teens during a critical phase of social-emotional development and the rising number of individual cases of youth recruitment and radicalization making headlines in the US (Ramirez, 2023; Weil, 2023; Young, 2022), such exposure to extremist ideology does indeed raise public concern. What are the experiences of hate speech and hate-based harassment among adolescents in online game platforms? How do teens and young adults perceive the danger of hate speech and how do they respond to it when encountered? Is there evidence to suggest that online games normalize hate among adolescents? Or is exposure to hate a potential normalizing variable? This paper details an exploratory study of the prevalence and potential normalization of hate speech and hate-based harassment in online games among adolescents and young adults. Following the United Nations (Baker, 2008), we define hate speech as “offensive discourse targeting a group or an individual based on inherent characteristics (such as race, religion or gender) and that may threaten social peace” (p.1) and, building on previous research conducted by the Anti-Defamation League (Anti-Defamation League, 2021, 2022a, 2024), we define hate-based harassment as “harassment targeting marginalized people because of their identity... typically for someone's gender, race or ethnicity, religion, sexual orientation, gender identity, physical appearance, or disability” (Anti-Defamation League, 2022b, p. 4). Here, we review the relevant literature on hate speech and hate-based harassment on online game platforms, the prevalence and developmental role of such social platforms for adolescents, and the definitions and mechanisms of normalization of otherwise extremist behavior and ideas. We then detail the participants in this study, the survey instrument and procedures used, our findings, and their import in light of the current literature on extremism in online games.

## 2 Literature review

Over the last 30 years, videogames have become a dominant form of entertainment with North America accounting for roughly one-quarter of the global market (Clement, 2023). Online video games in particular went mainstream during the global COVID-19 pandemic of 2020 as families adopted such platforms as a new “digital playground” for social interaction and joint activity among peers at a time when many were isolated at home (Kelly, 2021). Today, an estimated 40% of the global population plays games online (Baker, n.d.) with online games market revenues predicted to reach 27.97 billion USD in 2024 with an annual growth rate of 5.2% (Statista, 2023).

One key demographic subgroup within this large and growing online player base is “Gen Z” players or adolescent players between the ages of 13-25, with 27% of Gen Z teens (ages 14-19) and 21% of Gen Z young adults (ages 20-25) identifying gaming as their number one form of entertainment (Auxier and Patterson, 2022). In this work, we define “adolescents” as individuals of 13-25 years of age including both teens and young adults, in keeping with research findings on the neural changes that occur across this entire window of development, the earlier onset of biological changes marked by puberty, and the sociological shifts toward later shifts in responsibility that mark adulthood (Jaworska and MacQueen, 2015). During this crucial window of development, young people undergo significant change in terms of their identity formation (Klimstra, 2013), peer relationships (Brown, 2004), emotional regulation (Gupta and Gehlawat, 2020), and moral reasoning (Eisenberg and Morris, 2004). For those adolescents who game, online platforms such as Fortnite (Epic Games, 2017), Minecraft (Studios, 2011), League of Legends (Riot Games, 2013), or Valorant (Riot Games, 2020) function as third places (Steinkuehler and Williams, 2006), providing a context for social interaction and engagement beyond home and school (or work). Third places are characteristically more diverse than first or second places, offering the potential for exposure to new ideas and people that can expand one's social and intellectual circles or, in the case of extremism, its opposite.

Extremism in online games is particularly troubling given the nature of social bond formation in such spaces (Koehler et al., 2023; Schlegel and Amarasingam, 2022), the challenges game companies have in providing sufficient moderation (Jiang et al., 2019; Kou and Gui, 2021; Tekinbaş et al., 2021), and the significant presence of younger players (Clement, 2022; Lenhart, 2015). Recent research documents the rise of extremism in online game platforms for recruitment (Institute for Economics and Peace, 2022), community engagement with white separatist ideas (King and Leonard, 2016; Vaux et al., 2021), reinforcement of existing ideology among adherents (Lakhani, 2021), the spread of hate-based propaganda (Davey, 2021; Robinson and Whittaker, 2020), and concerns as to whether such platforms might serve to normalize otherwise radical beliefs (D'Anastasio, 2021). For a full review of the literature on, see Wells et al. (2024) and Steinkuehler and Squire (2024).

*Normalization* is a social process through which particular ideas and actions come to be seen as natural or normal in everyday life (Horwitz, 2016). In the context of extremist ideology, normalization occurs when ideas or behaviors once considered extreme, radical, or fringe are familiarized to the point in which they no longer provoke prohibition, perceptions of danger, or negative reactions from others. “When beliefs are shared by others, the idiosyncratic can become normalized” (Pierre, 2001). In the context of online gameplay, joint activity can create a sense of belonging and group membership that can be readily leveraged by extremist groups looking to inculcate new members into their folds (Koehler et al., 2023). Joint play in online games engenders a kind of “band of brothers” effect (Whitehouse et al., 2014) through joint struggle and triumph over shared challenge and conflict. Extremist groups use just such dynamics to build community and bonds among peers (Schlegel and Amarasingam, 2022). Previous survey studies provide examples of potential factors slowly shaping this normalization within gaming spaces, such as internalized misogyny (McCullough et al., 2020) among female gamers, minority gamers' exposure to racism, and association with the “gamer” identity (De Grove et al., 2015) all being heavily correlated with higher frequency of play. Furthermore, experiencing hateful conduct often leads to “desensitization” (Ortiz, 2019) and withdrawal (Fox and Tang, 2017) by the afflicted groups. In this way, in-game interactions may serve to enculturate players (Steinkuehler et al., 2012) into particular ideologies beyond the games themselves.

## 3 This study

This study aims to examine the frequency at which adolescent players encounter hate speech and hate-based harassment during online play, how they perceive and respond to such rhetoric, and the extent to which those encounters might be normalizing such hateful rhetoric and ideologies in online gaming spaces. Through an online survey, we attempt to answer the following research questions:

RQ1: What is the rate adolescent players are exposed to hate speech in online games and how do they perceive and respond to those encounters?

RQ2: At what rate do adolescent players become bystanders, victims, or perpetrators of hate-based harassment in online games? Does this vary by type of player?

RQ3: Does exposure to hate or particular gameplay habits lead to normalizing hate?

To answer RQ1, we ask players how often they come across hate speech during online play as well as how dangerous they perceive such statements to be and how they typically respond. We examine whether those responses work to perpetuate or productively combat hateful behavior and look for differences due to demographics. For RQ2, we ask players how often they encounter (as bystanders or victims) or initiate hate-based harassment targeting marginalized people because of their identity. We then test for differences due to demographics and, in the case of perpetration, personality traits or gameplay motivations. Finally, to tackle RQ3, we investigate the associations between gameplay habits (amount of gameplay, length of gameplay, gamer identity, perceived expertise, and frequency of competition) and exposure to hate (both speech and harassment) and three operational definitions of normalizing hate: (a) the frequency of perpetration of hate-based harassment, (b) diminished perceptions of the dangers of hate speech (both generally and specific forms of hate speech), and (c) perpetuating responses to hate online when it is encountered.

If successful, this study will inform public concerns as to the potential for online games - and/or the player behavior currently allowed largely unchecked within them - to normalize hate-based speech and behaviors among adolescent players. It will also provide further information on the prevalence of hateful conduct in online games among young people ages 13-25, current attitudes and responses toward it, and which demographic factors may leave some players at greater risk than others, potentially suggesting how players might themselves effectively and productively respond to such conduct in order to reduce its occurrence and to make online games safer for everyone.

## 4 Methods

### 4.1 Participants

A total of 602 participants 13–25 years of age were recruited via universities and high schools primarily (but not exclusively) in the coastal southwest of the United States. The average age of the resulting sample was 19.87 years (SD=1.77) enrolled in high school or college. The majority of participants were of Asian descent (60.1%), with Caucasian (18.3%) and Hispanic (12.0%) as the other most frequent ethnicities reported. The majority of respondents identified as male (66.1%) and heterosexual (74.6%). 4.0% report having a disability. Of the five personality traits measured for this study, the sample was generally high on positive personality traits, such as emotional self-regulation, communication, and empathy, and it was low on the more negative traits impulsivity and narcissism.

**Figure 1:**
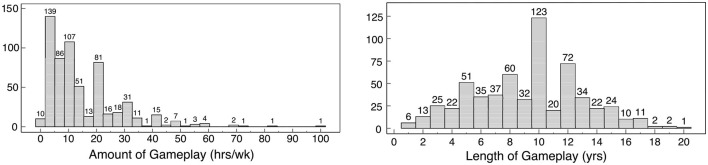
**(Left)** Participants' weekly amount of gameplay in hours and **(right)** gaming experience expressed in years.

The majority of the sample reported playing online games between 5 and 20 h per week and have been gaming for 8–12 years, as shown in Figure 1. The most frequently reported preferred online game genres were multiplayer online battle arenas (MOBAs), shooters, and sandbox games, all three of which frequently feature team-based competition, mechanics previously reported to show the highest reported rates of in-game hate speech and harassment (Anti-Defamation League, 2022a). 404 participants reported playing online games alone (67%), 511 with friends they met offline (85%), 315 with friends met online (52%), 159 with family (26%), and 170 with complete strangers (28%).

**Figure 2:**
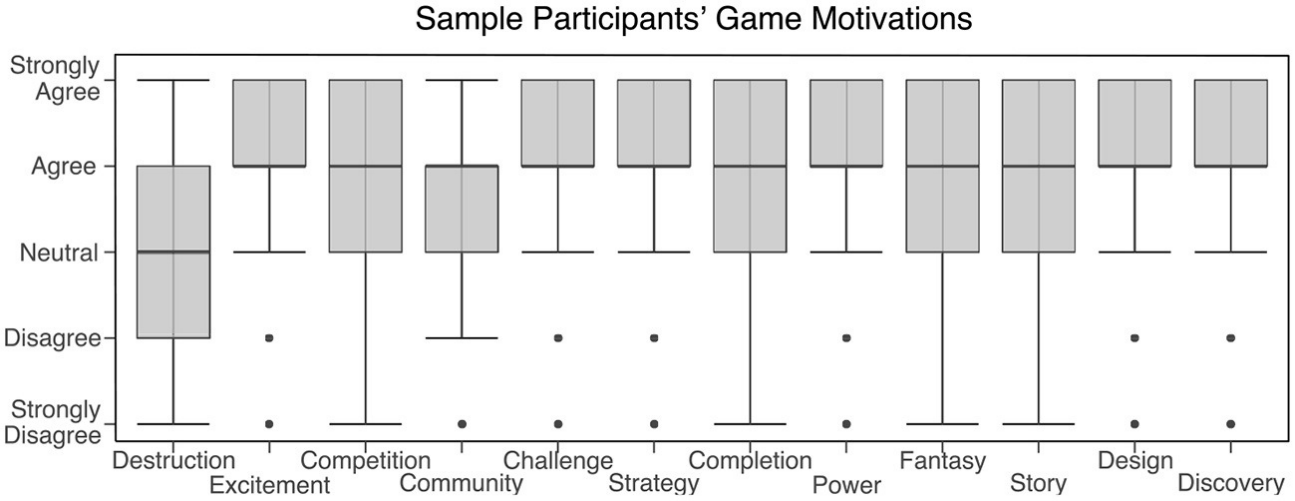
Reported motivations of gaming show a large diversity across the sample, covering the full spectrum reported in prior studies of online gameplay (Yee, 2006).

The majority of participants identify as “gamers” (72.6%) or describe themselves as at least moderately expert in the games they frequent (87.9%). Reported motivations for the game, shown in Figure 2, were diverse across the sample, covering the full gamut of those reported in prior studies of online gameplay (Yee, 2006).

### 4.2 Instrument

The survey instrument measures the frequency and duration of online gameplay and exposure to hate speech and hate-base harassment, attitudes toward hate speech, responses to such content, as well as basic demographic variables, such as gamer identity, self-perceptions of expertise, and frequency of competitive play. See Appendix A for a full list of survey items. Items related to hate speech were largely adopted from the Anti-Defamation League (2021) report on toxicity and harassment in games as well as the 2018 Cooperative Congressional Election Survey (Schaffner et al., 2019). The items included representing common racist or sexist talking points that were either prevalent at the time of data collection (e.g. “China knowingly released the COVID virus on the globe.”) or are longstanding cornerstones of extremist ideologies (“White genocide is real.”). The options provided as players' possible responses to such talking points were largely adapted from the ADL report (Anti-Defamation League, 2021) as well, along with a free response option intended to cover each individual's own experiences. They were then organized as either perpetuating (continuing/exacerbating the problem), productive (addressing the problem), or withdrawal (protecting oneself) responses.

Participants were asked to rate their experiences with hate-based harassment during play from the perspective of three different roles: bystanders, victims, and perpetrators. Originally applied in the context of genocide (Ehrenreich and Cole, 2005), recent research has examined these roles in events of harassment and cyberbullying (Jones et al., 2015). Participants were given a single multiple-response category for bystander experiences (“I have witnessed people being harassed in an online game based on…. (check all that apply)”) and for victim experiences (“I have been harassed in an online game based on my…. (check all that apply)”) with response selections including ethnicity, gender, sexual orientation, religion, and disability status.

Personality traits have been shown to correlate with one's propensity for toxic behaviors online (Hong and Cheng, 2018; Kordyaka et al., 2023). Traits like high extraversion and low agreeableness predict toxicity, while high narcissism, aggression, and machiavellianism predict hate speech instigation (ElSherief et al., 2018). To examine the potential influence personality might have on one's assessment of hate speech, we measured several factors, including impulsivity, empathy, narcissism, emotional self-regulation, and communication. The impulsivity and empathy assessments were taken from the Cyber-Aggression Questionnaire for Adolescents (Álvarez-García et al., 2016). The possible player motivations were adapted from Motivations for Play in Online Games (Yee, 2006). This was to account for any potential correlations between players' hate speech responses and their core goals for playing overall. Demographic factors measured in this instrument are age, gender, education level, sexual orientation, disability status, disability type, religion, and ethnicity. These were chosen both as potential moderating variables as well as being common characteristics often targeted by hate speech.

The instrument was predominantly comprised of items asking participants to rate on a 5-point Likert scale (with the midpoint of the scale neutral) the frequency with which they encountered specific forms of hate speech (such as “white genocide is real”) and hate-based harassment online as well as their attitudes (specifically, how dangerous they perceive it to be) and reactions to it (such as laughing or supporting the victim). Demographic questions and questions related to gameplay habits were fixed response items (for example, requiring numeric input for age or number of years one has gamed). For items that were particularly sensitive, such as those asking participants to report their own perpetrator behavior, a text prompt was given immediately before the item that stated “Reminder: No identifying information associated with your survey responses are recorded, so all your answers are completely anonymous”.

Principal component analyses were used to validate the instrument using pilot data (n=300); Analyses confirmed that the underlying components measured by the instrument were indeed those intended, with Cronbach's alpha on all sets of variables over 0.66 (and on all but one over 0.75) and on all variables taken together 0.94.

### 4.3 Procedures

Participants were recruited through fliers shared during undergraduate lectures at the host university and online, describing the survey as a “research study examining negative experiences in online gaming environments”. Volunteers first completed a preliminary screening survey verifying their age and participation in online games, then completed consent forms (and assent forms in the case of participants under the age of 18) before participating in the investigation. The emails of all volunteers were then individually verified before the survey was administered to ensure the data collected were trustworthy.

The survey instrument was administered online via Qualtrics and took 15–20 min on average to complete with $10 digital gift cards sent via email as compensation for their time. Data collection took place between March and June 2022, during a tumultuous period within the United States. Society as a whole was still recovering from the peak of the COVID-19 pandemic, while the ramifications of the January 6th, 2021 attack on the Capitol were still being felt in a tense political landscape.

A total of n=602 participants took part in the online survey, resulting in a 95% confidence level and ±4% margin of error on most variables. Data were downloaded to a shared server, cleaned, removing data that were incomplete or whose participant email could not be verified, and then analyzed in R Statistical Computing Software. Bonferroni corrections were applied to all families of tests conducted to control for Type I errors across multiple comparisons, where a “family of tests” is simply defined as those relating to a single hypothesis (Lakens, 2020; Bender and Lang, 2001). See Appendix B for a full list of hypotheses tested and the concomitant alpha correction used for all tests related to that hypothesis.

## 5 Findings

### 5.1 RQ1 exposure, perception, and responses to hate speech

#### 5.1.1 Exposure to hate speech and perceived dangerousness

Participants were given example statements such as “white genocide is real” representing six different forms of hate speech: misogyny, racism, white supremacy, antisemitism, anti-Muslim, and anti-Asian. First, participants rated the frequency with which they encountered such statements in online gameplay using a Likert scale ranging from 1 (never) to 5 (always). Then they rated how dangerous they perceived these examples of hate speech statements from 1 (harmless) to 5 (very dangerous). Hate speech types with more than one item were then combined into index composite variables.

More than 84.9% of participants reported encountering some form of hate speech in online games. Misogyny, Anti-Muslim, and anti-Asian hate speech were the most prevalent forms encountered. The average exposure to hate speech across all types was 1.9 (SD = 0.8) or rarely on the original five-point Likert scale. The average composite dangerousness rating across all types was 3.8 (SD = 0.8) or moderately dangerous on the original five-point Likert scale.

**Figure 3:**
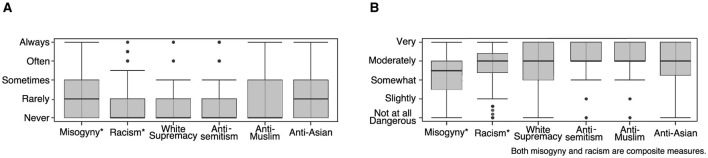
**(A)** Frequency of exposure to hate speech by type. **(B)** Perceived dangerousness of hate speech by type.

Figures 3A, B show the relative frequency of hate speech by type and the median and interquartile range for the perceived danger of each type of hate speech measured.

##### 5.1.1.1 Group differences

One-way analysis of variance (ANOVA) and Kruskal Wallis tests (for ordinal variables) were conducted to examine the effect of age, gender, education, and sexual orientation on overall perception of the dangers of hate speech (both generally and by type). The Games Howell test with alpha corrections was used for *post hoc* comparisons for all interval dependent variables and the Dunn-Bonferroni test was used for *post hoc* comparisons for all ordinal dependent variables.

###### 5.1.1.1.1 Age

Participants were partitioned into three age groups representing minors (14–17 years of age), college-age (18–21 years of age), and young adults (22–25 years of age). and their educational attainment was represented by four groups: some high school/GED, some college, bachelor's degree, and some graduate school/graduate degree. Neither age nor education was found to have a significant effect on the perceived dangers of hate speech (Table 1). However, significant effects were found for gender and for sexual orientation.

**Table 1:** Effects of age on perceived dangerous of hate speech, both generally and by type.

				**14–17 years old**		**18–21 years old**		**22–25 years old**	
	**F**_(2,599)_	***p*** **.adj**	$\eta_{p}^{2}$	**M**	**SD**	**M**	**SD**	**M**	**SD**
Overall perceived dangerousness of hate-speech (composite)	1.83	>0.999	0.006	3.5_*a*_	0.7	3.9_*a*_	0.8	3.9_*a*_	0.8
Perceived dangerousness of misogyny	4.90	0.055	0.002	2.6_*a*_	1.1	3.4_*a*_	1.0	3.4_*a*_	1.0
Perceived dangerousness of racism	1.39	>0.999	0.005	3.6_*a*_	0.6	3.9_*a*_	0.8	3.9_*a*_	0.8
	*H*(2)	***p*** **.adj**	ε^2^	**Mdn**	**IQR**	**Mdn**	**IQR**	**Mdn**	**IQR**
Perceived dangerousness of white supremacy	0.87	>0.999	−0.002	4.0_*a*_	1.0	4.0_*a*_	2.0	4.0_*a*_	2.0
Perceived dangerousness of anti-semitism	2.41	>0.999	0.001	4.0_*a*_	1.5	4.0_*a*_	1.0	4.5_*a*_	1.0
Perceived dangerousness of anti-Muslim	2.25	>0.999	<0.001	4.0*a*	0.5	4.0_*a*_	1.0	4.0_*a*_	1.0
Perceived dangerousness of anti-Asian	2.50	>0.999	<0.001	4.0_*a*_	2.0	4.0_*b*_	1.0	4.0_*a*_	1.0

Means/medians in a cell not sharing subscripts are significantly different from one another.

###### 5.1.1.1.2 Gender

One-way ANOVA tests and Kruskal Wallis tests (for ordinal dependent variables) revealed significant effects of gender on perceptions of the dangerousness of hate speech overall and on all six specific types of hate speech examined except white supremacy. The Games Howell test with alpha corrections was used for *post hoc* comparisons for all interval dependent variables and the Dunn-Bonferroni test was used for *post hoc* comparisons for all ordinal dependent variables (Table 2). Male individuals perceive hate speech in general as significantly *less dangerous* than females (*p* < 0.001) and nonbinary individuals (*p* < 0.001) do. Males also perceive specific types of hate speech as *less dangerous* than females and nonbinary individuals perceive it to be, namely: racist statements (*p* < 0.001, *p* < 0.001 respectively), and anti-Muslim statements (*p* = 0.010, *p* = 0.001 respectively). Males also perceive misogynistic (*p* = 0.002), antisemitic (*p* = 0.001), and anti-Asian (*p* = 0.006) hate speech as significantly *less dangerous* than females perceive it to be. Female individuals, however, perceive hate speech in general (*p* = 0.037) and antisemitic (*p* = 0.048) statements in particular as significantly *less dangerous* than nonbinary individuals do.

**Table 2:** Effects of gender on perceived dangerous of hate speech, both generally and by type.

				**Male**		**Female**		**Nonbinary**		**Prefer not to answer**	
	**F**_(3,595)_	***p*** **.adj**	$\eta_{p}^{2}$	**M**	**SD**	**M**	**SD**	**M**	**SD**	**M**	**SD**
Overall perceived dangerousness of hate-speech (composite)	8.73	<0.001	0.04	3.8_*a*_	0.8	4.0_*b*_	0.7	4.3_*c*_	0.5	4.2_*a,b,c*_	0.7
Perceived dangerousness of misogyny	6.12	0.003	0.03	3.2_*a*_	1.0	3.5_*b*_	1.0	3.7_*ab*_	1.1	3.8_*ab*_	0.8
Perceived dangerousness of racism	7.59	<0.001	0.04	3.8_*a*_	0.9	4.0_*b*_	0.7	4.3_*b*_	0.6	4.0_*ab*_	0.7
	*H*(3)	***p*** **.adj**	ε^2^	**Mdn**	**IQR**	**Mdn**	**IQR**	**Mdn**	**IQR**	**Mdn**	**IQR**
Perceived dangerousness of white supremacy	8.74	0.231	0.010	4.0_*a*_	1.75	4.0_*a*_	2.0	4.0_*a*_	1.0	4.0_*a*_	1.0
Perceived dangerousness of anti-semitism	20.08	0.001	0.029	4.0_*a*_	2.0	4.0_*b*_	1.0	5.0_*c*_	0.0	5.0_*a,b,c*_	0.75
Perceived dangerousness of anti-Muslim	20.00	<0.001	0.033	4.0*a*	1.0	4.0_*b*_	1.0	5.0_*c*_	1.0	5.0_*a,b,c*_	1.0
Perceived dangerousness of anti-Asian	16.36	0.007	0.022	4.0_*a*_	1.0	4.0_*b*_	2.0	5.0_*a,b*_	1.0	4.5_*a,b*_	1.0

Means/medians in a cell not sharing subscripts are significantly different from one another.

###### 5.1.1.1.3 Education

Given our sample demographics, educational attainment was represented by four groups: some high school/GED, some college, a bachelor's degree, and some graduate school/graduate degree. There were no significant effects of education on the perceived dangerousness of hate speech overall or by type (Table 3).

**Table 3:** Effects of education on perceived dangerous of hate speech, both generally and by type.

				**Some high school/ GED**		**Some college**		**Bachelor's degree**		**Some graduate school/ graduate degree**	
	**F**_(3,554)_	***p*** **.adj**	${\eta^{2}}_{p}$	**M**	**SD**	**M**	**SD**	**M**	**SD**	**M**	**SD**
Overall perceived dangerousness of hate-speech (composite)	2.24	0.577	0.01	3.7_*a*_	0.9	3.9_*a*_	0.8	3.9_*a*_	0.8	3.5_*a*_	0.7
Perceived dangerousness of misogyny	1.46	>0.999	0.007	3.2_*a*_	1.2	3.3_*a*_	1.0	3.5_*a*_	0.9	3.4_*a*_	0.9
Perceived dangerousness of racism	1.33	>0.999	0.007	3.8_*a*_	0.9	3.9_*a*_	0.8	3.9_*a*_	0.8	3.6_*a*_	0.7
	*H*(3)	***p*** **.adj**	ε^2^	**Mdn**	**IQR**	**Mdn**	**IQR**	**Mdn**	**IQR**	**Mdn**	**IQR**
Perceived dangerousness of white supremacy	8.67	0.238	0.009	4.0_*a*_	2.0	4.0_*a*_	2.0	4.0_*a*_	1.75	3.0_*a*_	1.5
Perceived dangerousness of anti-semitism	7.075	0.360	0.008	4.0_*a*_	2.0	4.0_*a*_	1.0	4.0_*a*_	1.0	4.0_*a*_	1.0
Perceived dangerousness of anti-Muslim	6.79	0.552	0.006	4.0*a*	2.0	4.0_*a*_	1.0	4.0_*a*_	1.0	4.0_*a*_	0.5
Perceived dangerousness of anti-Asian	5.63	0.916	0.004	4.0_*a*_	2.0	4.0_*a*_	1.0	4.0_*a*_	1.0	4.0_*a*_	0.5

Means/medians in a cell not sharing subscripts are significantly different from one another.

###### 5.1.1.1.4 Sexual orientation

One-way ANOVA tests and Kruskal Wallis tests (for ordinal dependent variables) revealed significant effects of sexual orientation on perceptions of the dangerousness of hate speech overall and three of six specific types of hate speech: antisemitism, anti-Muslim, and anti-Asian hate speech. The Games Howell test with alpha corrections was again used for *post hoc* comparisons for all interval dependent variables and the Dunn-Bonferroni test was again used for *post hoc* comparisons for all ordinal dependent variables (Table 4). Heterosexual individuals perceive hate speech in general as significantly *less dangerous* than asexual (*p* = 0.022) and bisexual individuals (*p* < 0.001) do. Heterosexual individuals also perceive specific types of hate speech as *less dangerous* than asexual and bisexual individuals perceive it to be, including antisemitism hate speech (*p* = 0.004, *p* = 0.020, respectively) and anti-muslim speech (*p* = 0.033, *p* = 0.001, respectively). Heterosexuals also perceive anti-Asian (*p* = 0.001) statements as significantly *less dangerous* than bisexual individuals do.

**Table 4:** Effects of sexual orientation on perceived dangerous of hate speech, both generally and by type.

				**Asexual**		**Bisexual**		**Heterosexual**		**Homosexual**	
	**F**_(3,554)_	***p*** **.adj**	$\eta_{p}^{2}$	**M**	**SD**	**M**	**SD**	**M**	**SD**	**M**	**SD**
Overall perceived dangerousness of hate-speech (composite)	6.44	0.002	0.03	4.2_*a*_	0.6	4.2_*a*_	0.6	3.8_*b*_	0.8	3.9_*a,b*_	0.9
Perceived dangerousness of misogyny	1.80	>0.999	0.010	3.5_*a*_	0.8	3.6_*a*_	1.0	3.3_*a*_	1.0	3.4_*a*_	0.9
Perceived dangerousness of racism	4.04	0.051	0.020	4.1	0.8	4.1_*a*_	0.7	3.8_*a*_	0.8	4.0_*a*_	0.8
	**H(3)**	***p*** **.adj**	ε^2^	**Mdn**	**IQR**	**Mdn**	**IQR**	**Mdn**	**IQR**	**Mdn**	**IQR**
Perceived dangerousness of white supremacy	9.42	0.170	0.011	4.0_*a*_	2.0	4.0_*a*_	1.25	4.0_*a*_	1.0	4.0_*a*_	2.25
Perceived dangerousness of anti-semitism	20.47	0.001	0.029	5.0_*a*_	0.0	5.0_*a*_	1.0	4.0_*b*_	2.0	5.0_*a,b*_	1.0
Perceived dangerousness of anti-Muslim	19.96	0.001	0.028	5.0_*a*_	1.0	5.0_*a*_	1.0	4.0_*b*_	1.0	4.0_*a,b*_	1.0
Perceived dangerousness of anti-Asian	19.58	0.001	0.028	5.0_*a*_	1.0_*a,b*_	5.0_*a*_	1.0	4.0_*b*_	2.0	4.5_*a,b*_	2.0

Means/medians in a cell not sharing subscripts are significantly different from one another.

#### 5.1.2 Responses to hate speech

**Figure 4:**
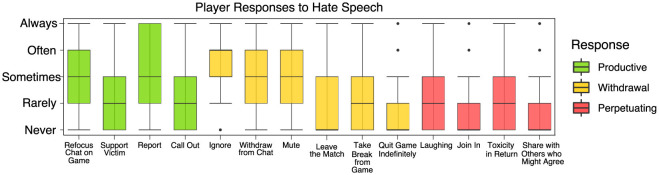
Player responses to hate-speech, productive, withdrawal, and perpetuating responses are denoted with green, yellow, and red colors, respectively.

How do players typically respond to hate speech in online games? Survey participants were asked to rate 14 common responses to hate speech in online games (Jubany, 2015; Passmore and Mandryk, 2020) in terms of the frequency with which they engaged in each type. The common responses used as prompts fell into three broad categories. Productive responses are those that overtly signal within the immediate context of the event that the hate-based statement is not acceptable and not tolerated. Withdrawal responses are those in which the player ceases to participate in some way. Such responses are useful for strategies for removing oneself from harm's way but stop short of signaling that hate speech is outside the bounds of what's acceptable or normal. Perpetuating responses are those that escalate the situation, either by encouraging hate speech or even retaliating in kind. Figure 4 shows their respective self-reported frequencies.

An index measure was constructed for each type—productive responses (green in Figure 4), withdrawal responses (yellow in Figure 4), and perpetuating responses (red in Figure 4)—by averaging across all variables within the category. One-way repeated measures ANOVA found significant differences in the frequency with which players engage in each type of response to hate speech [F_(2,1188)_ = 217.73, *p* < 0.001]. Pairwise *post hoc t*-test comparisons with Bonferroni corrections showed that participants engaged in productive (M = 2.6, SD = 0.9) and withdrawal responses (M = 2.5, SD = 0.7) [t(575) = 2.77, p = 0.018] to hate speech significantly more frequently than perpetuating responses (M = 1.8, SD = 0.7) to hate speech (t(575) = 5.14, p < 0.001).

##### 5.1.2.1 Group differences

One-way analysis of variance (ANOVA) and Kruskal Wallis tests (for ordinal variables) were conducted to examine the effect of age, gender, education, and sexual orientation on responses to hate speech (by type and specific response). Significant effects were found for age, gender, and sexual orientation.

###### 5.1.2.1.1 Age

There were significant main effects of age on laughing in response to hate speech (Table 5). Dunn *post hoc* tests with Bonferroni alpha adjustments revealed that players between the ages of 14-17 report laughing significantly more frequently in response to hate speech than older players 18–21 years of age (p = 0.002) and 22–25 years of age (*p* = 0.002).

**Table 5:** Effects of age on responses to hate speech, by type and specific response.

				**14–17 years old**		**18–21 years old**		**22–25 years old**	
**Grouped responses**	**F**(2, 592 − 599)	***p*** **.adj**	$\eta_{p}^{2}$	**M**	**SD**	**M**	**SD**	**M**	**SD**
Productive responses	0.40	>0.999	0.001	2.5_*a*_	0.7	2.6_*a*_	0.9	2.7_*a*_	0.8
Withdrawal responses	0.80	>0.999	0.003	2.4_*a*_	0.5	2.5_*a*_	0.7	2.4_*a*_	0.7
Perpetuating responses	2.26	>0.999	0.008	2.2_*a*_	0.6	1.8_*a*_	0.7	1.8_*a*_	0.7
**Responses**	**H(2)**	***p*** **.adj**	ε^2^	**Mdn**	**IQR**	**Mdn**	**IQR**	**Mdn**	**IQR**
Refocus chat on game	0.20	>0.999	−0.003	3.0_*a*_	2.5	3.0_*a*_	2.0	3.0_*a*_	2.0
Support victim	0.50	>0.999	−0.003	2.0_*a*_	1.0 2.0_*a*_	2.0	1.5_*a*_	2.0
Report	2.80	>0.999	0.001	2.0_*a*_	2.0	3.0_*a*_	3.0	3.5_*a*_	3.0
Call out speaker	0.672	>0.999	−0.002	2.0_*a*_	1.0	2.0_*a*_	2.0	2.0_*a*_	1.75
Ignore it	1.01	>0.999	−0.002	4.0_*a*_	3.0	4.0_*a*_	1.0	4.0_*a*_	1.8
Withdrawal from chat	0.67	>0.999	−0.002	3.0_*a*_	2.0	3.0_*a*_	2.0	3.0_*a*_	2.0
Mute the speaker	0.79	>0.999	−0.002	3.0_*a*_	2.0	3.0_*a*_	2.0	3.0_*a*_	2.0
Leave match	0.48	>0.999	−0.003	1.0_*a*_	1.0	1.0_*a*_	2.0	1.0_*a*_	2.0
Take break from game	1.51	>0.999	−0.001	2.0_*a*_	2.0	2.0_*a*_	2.0	1.0_*a*_	1.8
Quit game indefinitely	0.864	>0.999	−0.002	1.0_*a*_	0.5	1.0_*a*_	1.0	1.0_*a*_	1.0
Laughing	12.27	0.039	0.017	4.0_*a*_	2.0	2.0_*b*_	2.0	2.0_*b*_	2.0
Toxicity in return	0.27	>0.999	−0.003	2.0_*a*_	1.5	2.0_*a*_	2.0	2.0_*a*_	2.0
Join In	2.715	>0.999	0.008	1.5_*a*_	1.0	1.0_*a*_	1.0	1.0_*a*_	1.0
Share with others who might agree	1.601	>0.999	−0.001	1.0_*a*_	1.5	1.0_*a*_	1.0	1.0_*a*_	1.0
Other	6.681	0.638	0.008	1.0_*a*_	1.5	1.0_*a*_	2.0	3.5_*a*_	3.0

Means/medians in a cell not sharing subscripts are significantly different from one another.

###### 5.1.2.1.2 Gender

Gender has significant effects on responses to hate speech by type and by specific response. Games Howell test with alpha corrections and Dunn-Bonferroni test (for ordinal variables) were again used for *post hoc* comparisons (Table 6).

**Table 6:** Effects of gender on responses to hate speech, by type and specific response.

				**Male**		**Female**		**Nonbinary**		**Prefer not to answer**	
**Grouped responses**	**F**_(3, 588 − 595)_	***p*** **.adj**	$\eta_{p}^{2}$	**M**	**SD**	**M**	**SD**	**M**	**SD**	**M**	**SD**
Productive responses	1.32	>0.999	0.007	2.6	0.8	2.6	0.8	2.9	0.9	2.5	0.8
Withdrawal responses	8.17	<0.001	0.001	2.4_*a*_	0.7	2.7_*b*_	0.7	2.6_*a,b*_	0.8	2.7_*a,b*_	0.8
Perpetuating responses	7.22	0.002	0.040	1.9_*a*_	0.7	1.7_*b*_	0.6	1.7_*a,b*_	0.6	1.6_*a,b*_	0.7
**Responses**	**H(3)**	***p*** **.adj**	ε^2^	**Mdn**	**IQR**	**Mdn**	**IQR**	**Mdn**	**IQR**	**Mdn**	**IQR**
Refocus chat on game	3.19	>0.999	<0.001	3.0_*a*_	2.0	3.0_*a*_	2.0	3.0_*a*_	2.0	2.5_*a*_	2.5
Support victim	7.21	>0.999	0.007	1.0_*a*_	1.0	2.0_*a*_	2.0	2.0_*a*_	2.0	2.0_*a*_	2.0
Report	2.480	>0.999	−0.001	3.0_*a*_	3.0	3.0_*a*_	3.0	4.0_*a*_	2.0	3.5_*a*_	2.5
Call out speaker	5.864	>0.999	0.005	2.0_*a*_	2.0	2.0_*a*_	2.0	3.0_*a*_	2.0	2.0_*a*_	1.8
Ignore it	19.51	0.004	0.0276	4.0_*a*_	2.0	3.0_*b*_	2.0	3.0_*b*_	2.0	4.0_*a,b*_	0.8
Withdrawal from chat	14.29	0.0456	0.019	3.0_*a*_	2.0	4.0_*b*_	1.0	3.5_*a,b*_	1.0	3.5_*a,b*_	1.0
Mute the speaker	3.69	>0.999	0.001	3.0_*a*_	2.0	3.0_*a*_	1.0	3.0_*a*_	1.75	3.0_*a*_	1.0
Leave match	29.92	<0.001	0.045	1.0_*a*_	1.0	2.0_*b*_	2.0	2.0_*b*_	2.0	3.0_*a,b*_	1.8
Take break from game	37.51	<0.001	0.058	2.0	2.0	1.0	1.0	2.0	2.0	1.5	2.0
Quit game indefinitely	28.20	<0.001	0.042	1.0_*a*_	1.0	1.0_*b*_	0.0	1.0_*a,b*_	1.0	1.5_*a,b*_	1.0
Laughing	65.02	<0.001	0.104	2.0_*a*_	3.0	1.0*b*	1.0	1.0_*b*_	1.0	1.0_*a,b*_	1.0
Toxicity in return	4.35	>0.999	0.002	2.0*a*	2.0	2.0_*a*_	1.0	1.5_*a*_	1.8	1.0_*a*_	1.8
Join in	2.26	>0.999	−0.001	1.0*a*	1.0	1.0_*a*_	1.0	1.0_*a*_	1.0	1.0_*a*_	1.0
Share with others who might agree	6.74	>0.999	0.006	1.0_*a*_	1.0	1.0_*a*_	1.0	1.0_*a*_	1.0	1.0_*a*_	1.0
Other	3.13	>0.999	0.002	1.0	2.0	2.0	3.0	3.0	3.0	n/a	n/a

Means/medians in a cell not sharing subscripts are significantly different from one another.

In terms of response types, male individuals engage significantly more frequently in perpetuating responses than females (*p* < 0.001), while females engage significantly more frequently in withdrawal responses (*p* < 0.001) than males. Specifically, males more frequently laugh in response to hate speech than females (*p* < 0.001) and nonbinary individuals (*p* = 0.001); they also more frequently ignore hate speech than females (*p* = 0.001) and nonbinary individuals (*p* = 0.031). Females, however, are more likely to withdraw from in-game chat and take a break from the game (*p* < 0.001) in response to hate speech (*p* = 0.002) than males, and both females (*p* < 0.001) and nonbinary individuals (*p* = 0.002) are more likely to leave the match than males.

###### 5.1.2.1.3 Education

There were no significant effects of education on the responses to hate speech by type and by specific response (Table 7).

**Table 7:** Effects of education on responses to hate speech, by type and specific response.

				**Some high school/ GED**		**Some college**		**Bachelor's degree**		**Some graduate school/ graduate degree**	
**Grouped responses**	**F**_(3, 588 − 595)_	***p*** **.adj**	$\eta_{p}^{2}$	**M**	**SD**	**M**	**SD**	**M**	**SD**	**M**	**SD**
Productive responses	0.57	>0.999	0.003	2.6_*a*_	0.8	2.6_*a*_	0.9	2.7_*a*_	0.9	2.7_*a*_	0.7
Withdrawal responses	0.28	>0.999	0.001	2.5_*a*_	0.7	2.5_*a*_	0.7	2.5_*a*_	0.8	2.7_*a*_	0.6
Perpetuating responses	2.552	0.985	0.01	1.9_*a*_	0.7	1.8_*a*_	0.7	1.9_*a*_	0.6	2.1_*a*_	0.9
**Responses**	**H(3)**	***p*** **.adj**	ε^2^	**Mdn**	**IQR**	**Mdn**	**IQR**	**Mdn**	**IQR**	**Mdn**	**IQR**
Refocus chat on game	2.70	>0.999	<0.001	3.0_*a*_	2.0	3.0_*a*_	2.0	3.0_*a*_	2.0	3.0_*a*_	0.5
Support victim	1.86	>0.999	<0.001	2.0_*a*_	1.0	1.0_*a*_	2.0	2.0_*a*_	2.0	2.0_*a*_	2.0
Report	2.241	>0.999	−0.001	3.0_*a*_	3.0	4.0_*a*_	3.0	3.0_*a*_	3.0	4.0_*a*_	2.0
Call out speaker	2.053	>0.999	−0.002	2.0_*a*_	2.0	2.0_*a*_	2.0	2.0_*a*_	3.0	2.0_*a*_	1.5
Ignore it	2.87	>0.999	<0.001	4.0_*a*_	2.0	4.0_*a*_	1.0	4.0_*a*_	2.0	3.0_*a*_	1.0
Withdrawal from chat	1.95	>0.999	−0.002	3.0_*a*_	2.0	3.0_*a*_	2.0	3.0_*a*_	2.0	3.0_*a*_	0.5
Mute the speaker	2.11	>0.999	−0.001	3.0_*a*_	2.0	3.0_*a*_	2.0	3.0_*a*_	2.0	3.0_*a*_	0.0
Leave match	1.77	>0.999	−0.002	1.0_*a*_	1.0	1.0_*a*_	2.0	2.0_*a*_	2.0	2.0_*a*_	1.5
Take break from game	3.546	>0.999	0.001	2.0_*a*_	2.0	2.0_*a*_	2.0	2.0_*a*_	2.0	3.0_*a*_	1.5
Quit game indefinitely	6.155	>0.999	0.005	1.0_*a*_	1.0	1.0_*a*_	1.0	1.0_*a*_	1.0	2.0_*a*_	1.5
Laughing	9.44	0.433	0.0108	2.0_*a*_	3.0	2.0_*a*_	2.0	2.0_*a*_	2.0	2.0_*a*_	2.0
Toxicity in return	1.96	>0.999	<0.001	2.0_*a*_	2.0	2.0_*a*_	2.0	2.0_*a*_	2.0	2.0_*a*_	2.5
Join in	3.52	>0.999	<0.001	1.0_*a*_	1.0	1.0_*a*_	1.0	1.0_*a*_	1.0	1.0_*a*_	1.0
Share with others who might agree	6.80	>0.999	0.006	1.0_*a*_	1.0	1.0_*a*_	1.0	1.0_*a*_	1.0	2.0_*a*_	2.0
Other	1.95	>0.999	−0.002	2.0_*a*_	2.0	2.0_*a*_	3.0	1.0_*a*_	3.0	1.0_*a*_	0.0

Means/medians in a cell not sharing subscripts are significantly different from one another.

###### 5.1.2.1.4 Sexual orientation

One-way ANOVA tests and Kruskal Wallis tests (for ordinal dependent variables) revealed significant effects of sexual orientation on two specific responses to hate speech: laughing and taking a break from the game (Table 8). Dunn-Bonferroni *post hoc* tests revealed that heterosexual individuals more frequently laugh at hate speech than asexual (*p* = 0.027) and bisexual (*p* = 0.016) individuals and less frequently take a break from the game in response to hate speech than bisexual (*p* = 0.025) and homosexual individuals (*p* = 0.025).

**Table 8:** Effects of sexual orientation on responses to hate speech, by type and specific response.

				**Asexual**		**Bisexual**		**Hetero-sexual**		**Homo-sexual**	
**Grouped responses**	**F**_(3, 547 − 554)_	***p*** **.adj**	$\eta_{p}^{2}$	**M**	**SD**	**M**	**SD**	**M**	**SD**	**M**	**SD**
Productive Responses	3.45	0.297	0.02	2.9_*a*_	0.8	2.8_*a*_	0.8	2.6_*a*_	0.9	2.9_*a*_	0.9
Withdrawal Responses	1.89	>0.999	0.01	2.6_*a*_	0.8	2.6_*a*_	0.7	2.5_*a*_	0.7	2.6_*a*_	0.6
Perpetuating Responses	1.47	>0.999	0.008	1.8_*a*_	0.7	1.8_*a*_	0.7	1.9_*a*_	0.7	1.6_*a*_	0.7
**Responses**	**H(3)**	***p*** **.adj**	ε^2^	**Mdn**	**IQR**	**Mdn**	**IQR**	**Mdn**	**IQR**	**Mdn**	**IQR**
Refocus chat on game	2.44	>0.999	0.001	3.0*a*	2.0	3.0_*a*_	1.0	3.0_*a*_	2.0	3.0_*a*_	2.0
Support victim	10.50	0.266	0.013	2.0_*a*_	2.0	2.0_*a*_	2.0	1.0_*a*_	1.0	2.5_*a*_	2.0
Report	12.50	0.106	0.016	4.0_*a*_	2.0	4.0_*a*_	2.0	3.0_*a*_	3.0	4.0_*a*_	3.0
Call out speaker	6.35	>0.999	0.006	3.0_*a*_	2.0	2.5_*a*_	3.0	2.0_*a*_	2.0	2.5_*a*_	2.0
Ignore it	6.120	>0.999	0.005	4.0_*a*_	2.0	3.0_*a*_	2.0	4.0_*a*_	1.0	3.0_*a*_	1.3
Withdrawal from chat	9.53	0.414	0.011	4.0_*a*_	1.0	4.0_*a*_	1.0	3.0_*a*_	2.0	4.0_*a*_	1.0
Mute the speaker	1.52	>0.999	−0.002	3.0_*a*_	2.0	3.0_*a*_	1.0	3.0_*a*_	2.0	3.0_*a*_	2.0
Leave Match	12.93	0.09	−0.017	1.0_*a*_	2.0	2.0_*a*_	2.0	1.0_*a*_	1.0	2.0_*a*_	2.0
Take break from game	15.55	0.025	0.021	2.0_*a,b*_	1.0	2.0_*a*_	2.0	1.0_*a,b*_	2.0	2.0_*a*_	1.0
Quit game indefinitely	5.01	>0.999	0.003	1.0_*a*_	1.0	1.0_*a*_	1.0	1.0_*a*_	1.0	1.0_*a*_	1.0
Laughing	19.03	0.005	0.027	1.0_*a*_	1.0	2.0_*a*_	2.0	2.0_*b*_	3.0	1.0_*a,b*_	1.3
Toxicity in return	6.46	>0.999	0.006	1.0_*a*_	1.0	2.0_*a*_	2.0	2.0_*a*_	2.0	1.0_*a*_	1.0
Join in	0.227	>0.999	0.002	1.0_*a*_	1.0	1.0_*a*_	1.0	1.0_*a*_	1.0	1.0_*a*_	0.0
Share with others who might agree	1.59	>0.999	−0.002	1.0_*a*_	1.0	1.0_*a*_	1.0	1.0_*a*_	1.0	1.0_*a*_	1.0
Other	0.767	>0.999	−0.004	1.0_*a*_	2.0	3.0_*a*_	3.0	2.0_*a*_	3.0	2.0_*a*_	2.3

Means/medians in a cell not sharing subscripts are significantly different from one another.

### 5.2 RQ2. Bystander, victim, and perpetrator experiences of hate-based harassment

#### 5.2.1 Exposure to hate-based harassment

Participants were asked how often they were a bystander to or victim of in-game hate-based harassment (HBH), or harassment targeting an individual based on one's gender, sexual orientation, disability status, religion, or ethnicity.

##### 5.2.1.1 Bystanders

**Figure 5:**
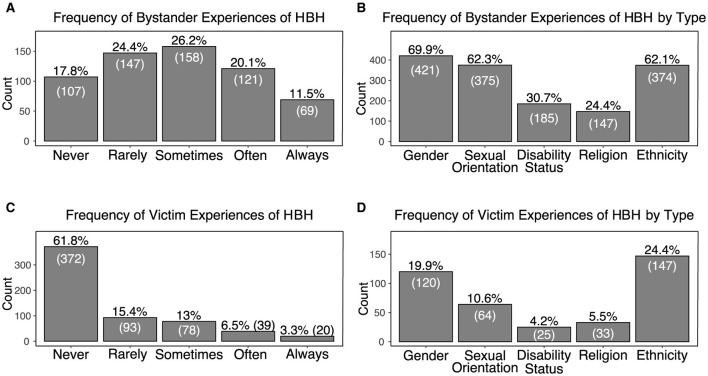
**(A)** Frequency of bystander exposure to HBH in online games, **(B)** Proportion of players who have been bystanders to HBH by type. **(C)** Frequency of victim exposure to HBH in online games **(D)** Proportion of players who have been victims to HBH by type.

Of the sampled adolescent players, 82.2% have been bystanders to HBH in online games. Across all types, the median of reported frequency of bystander exposure to HBH was 3.0 or sometimes (IQR=2). Among these, gender-based harassment was the most prevalent form witnessed (69.9% of all participants) followed by harassment based on sexual orientation (62.3%) and ethnicity (62.1%). Figures 5A, B show the frequency of bystander exposure to HBH and the proportion of players who have been bystanders to each of the five types of HBH examined.

##### 5.2.1.2 Victims

More than a third (38.2%) of participants reported being the victim of HBH in online games. The median of the reported frequency of victim exposure to HBH across all types was 1.0 or never (IQR=2). Ethnicity-based harassment is the most prevalent form of reported HBH victimization (24.4%) followed by harassment based on gender (19.9%) and sexual orientation (10.6%). Figure 5C shows the frequency of victim exposure to HBH and Figure 5D the proportion of players who have been victims to each type of HBH examined.

##### 5.2.1.3 Group Differences in Victimization

Kruskal-Wallis tests were conducted to compare the effect of each demographic variable on the frequency of HBH victimization, in general and across all targeted demographic types. We then tested for group differences within that demographic category to understand which specific subgroups were most at risk. For example, among those who have been victims of gender-based harassment, which genders are more frequently targeted?

###### 5.2.1.3.1 Gender

There were no significant effects of gender on hate-based harassment victimization across all types (Table 9). We next tested for differences in gender-based harassment by gender category to understand whether some gender groups may be more at risk for being a target for HBH. A chi-square test of independence found a significant relationship between a participant's gender identity category and the frequency of being a victim of gender-based harassment (Table 9). Examination of the standardized residuals (Sharpe, 2019) shows that the significant relationship found is primarily due to differences between male and female responses. Female individuals (r = 7.76) are significantly more likely to be victimized by gender-harassment than expected based on overall proportions and male individuals (r = −8.811) are significantly less likely to be victimized based on gender.

Table 9Kruskal-Wallis test results for effects of different demographics on HBH victimization.

**Table:** 

**Gender**			**Male**		**Female**		**Nonbinary**		**Prefer not to answer**	
H(3)	*p*.adj	ε^2^	Mdn	IRQ	Mdn	IRQ	Mdn	IRQ	Mdn	IRQ
4.66	0.397	0.003	1.0_*a*_	1.0	1.0_*a*_	2.0	1.0_*a*_	1.8	1.5_*a*_	1.0
**Sexual Orientation**			**Asexual**		**Bi-sexual**		**Hetero-sexual**		**Homo-sexual**	
H(3)	*p*.adj	ε^2^	Mdn	IRQ	Mdn	IRQ	Mdn	IRQ	Mdn	IRQ
15.24	0.003	0.021	2.0_*a*_	3.0	1.5_*a*_	2.0	1.0_*a*_	1.0	2.0_*a*_	2.0
**Disability Status**					**Disability**		**No Disability**		**Prefer not to answer**	
H(3)	*p*.adj		ε^2^		Mdn	IRQ	Mdn	IRQ	Mdn	IRQ
7.56	0.0456		0.009		2.0_*a*_	2.5	1.0_*b*_	1.0	1.0_*a*_, *b*	1.0

**Table:** 

**Religion**			**Agnostic/ Atheist**		**Buddhist**		**Catholic**		**Christian**		**Hindu**		**Islamic**		**Jewish**	
H(3)	*p*.adj	ε^2^	Mdn	IRQ	Mdn	IRQ	Mdn	IRQ	Mdn	IRQ	Mdn	IRQ	Mdn	IRQ	Mdn	IRQ
2.07	>0.999	−0.007	1.0_*a*_	1.0	1.0_*a*_	1.0	1.5_*a*_	1.8	1.0_*a*_	2.0	1.0_*a*_	1.8	1.0_*a*_	2.0	1.0_*a*_	1.0

**Table:** 

**Ethnicity**					**Asian**			**Black/ African**			**White**			**Hispanic/ Latinx**		
H(3)	*p*.adj		ε^2^		Mdn		IRQ	Mdn		IRQ	Mdn		IRQ	Mdn		IRQ
9.78	0.041		0.018		1.0_*a*_		1.0	3.0_*b*_		3.0	1.0_*a*_		1.0	1.0_*a*_		1.0

Medians in a cell not sharing subscripts are significantly different from one another.

###### 5.2.1.3.2 Sexual orientation

The Kruskal-Wallis test revealed significant effects of sexual orientation on overall HBH victimization. Dunn *post hoc* tests with Bonferroni corrections, however, show no significant differences among sexual orientation categories. The largest group difference in medians is between bisexual individuals (Mdn = 1.5, IQR = 2.0) and heterosexual individuals (Mdn = 1.0, IQR = 1.0), yet it does not rise to the level of significance (*p*.adj = 0.076) (Table 9).

A chi-square test of independence found a significant relationship between a participant's sexual orientation and HBH victimization based on sexual orientation (Table 10). From the standardized residuals we find that this significant relationship is due to differences among all four sexual orientation categories: *Asexual* (r = 2.23), *bisexual* (r = 3.40) and *homosexual* individuals (r = 4.39) are *more likely* to be the victim of hate-based harassment on the basis of sexual orientation while *heterosexual individuals* (r = −6.07) are *less* likely to be victimized in this way.

**Table 10:** Effects of demographic categories on the corresponding hate-based harassment victimization type.

					**Male**	**Female**	**Non-binary**	**Prefer not to answer**	**χ^2^(3)**	***p*.adj**
**Gender-based harassment victimization**										
Yes	Expected				79.7	33.1	5.2	2.0	77.61	<0.001
	Observed				39	67	10	4		
	Standardized Residuals				(−8.81)	(7.76)	(2.40)	(1.59)		
No	Expected				318.3	131.9	20.8	8.0		
	Observed				359	98	16	6		
	Standardized residuals				(8.81)	(−7.76)	(−2.40)	(−1.59)		
					**Asexual**	**Bisexual**	**Hetero-sexual**	**Homo-sexual**	χ^2^(3)	***p*** **.adj**
**Sexual orientation-based harassment victimization**										
Yes	Expected				2.6	6.3	47.5	2.5	40.69	<0.001
	Observed				6	14	30	9		
	Standardized residuals				(2.23)	(3.40)	(−6.07)	(4.39)		
No	Expected				22.4	53.7	401.5	21.5		
	Observed				19	46	419	15		
	Standardized residuals				(−2.23)	(−3.40)	(6.07)	(−4.39)		
						**Disability**	**No disability**	**Prefer not to answer**	χ^2^(3)	***p*** **.adj**
**Disability-based harassment victimization**										
Yes	Expected					1.0	22.8	1.2	39.30	<0.001
	Observed					7	17	1		
	Standardized residuals					(6.27)	(−4.24)	(−0.16)		
No	Expected					23.0	527.2	26.8		
	Observed					17	533	27		
	Standardized residuals					(−6.27)	(4.24)	(0.16)		
		**Agnostic/ atheist**	**Buddhist**	**Catholic**	**Christian**	**Hindu**	**Muslim**	**Jewish**	χ^2^(3)	***p*** **.adj**
**Religion-based harassment victimization**										
Yes	Expected	14.4	3.1	0.8	8.6	0.8	1.0	0.3	37.03	<0.001
	Observed	7	2	1	12	0	6	1		
	Standardized residuals	(−2.84)	(−0.68)	(0.19)	(1.44)	(−0.96)	(5.42)	(1.33)		
No	Expected	227.6	48.9	13.2	135.4	13.2	15.1	4.7		
	Observed	235	50	13	132	14	10	4		
	Standardized residuals	(2.84)	(0.68)	(−0.19)	(−1.44)	(0.96)	(−5.42)	(−1.33)		
					**Asian**	**Black/ African**	**White**	**Hispanic/ Latinx**	*X*^2^(3)	***p*** **.adj**
**Ethnicity-based harassment victimization**										
Yes	Expected				86.8	3.6	26.4	17.3	24.30	<0.001
	Observed				97	8	9	20		
	Standardized residuals				(2.12)	(2.70)	(−4.33)	(0.81)		
No	Expected				275.2	11.4	83.6	54.7		
	Observed				265	7	101	52		
	Standardized residuals				(−2.12)	(−2.70)	(4.33)	(−0.81)		

###### 5.2.1.3.3 Disability status

The test found significant effects of disability status on hate-based harassment victimization generally (Table 9). Dunn post-hoc tests with Bonferroni corrections revealed that individuals who identify as having a disability are significantly more likely the victim of hate-based harassment in general (Mdn = 2.0, IQR = 2.5) than individuals who do not identify as having a disability (Mdn = 1.0, IQR = 1.0) (*p*.adj = 0.002).

A chi-square test of independence was again used to check for differences among subgroups in their risk for HBH victimization based specifically on disability status, finding a significant relationship between a participant's disability status and being victimized on the basis of disability status (Table 10). From the standardized residuals, we find that individuals with a disability are significantly *more likely* to be victimized by disability-based harassment (r = 6.27) and individuals without a disability are significantly *less likely* to be harassed as such (r = −4.24).

###### 5.2.1.3.4 Religion

Religion. The Kruskal-Wallis test revealed no significant effects of religion on hate-based harassment victimization generally. Table 9 details the statistical results.

We then tested for differences by type of religion in religion-based harassment using a chi-square test of independence; we found a significant relationship between religious affiliation and religion-based HBH (Table 10). From the standardized residuals, we find that this significant relationship is primarily due to differences found in Agnostic/Atheist and Muslim responses. Agnostics/Atheists (r = 2.84) are less likely to be victimized by religious-based harassment than expected based on overall proportions and Muslims (r = 5.42) are significantly more likely to be victimized by religious-based harassment than expected based on overall proportions.

###### 5.2.1.3.5 Ethnicity

The test revealed significant effects of ethnicity on HBH victimization generally (across all types) (Table 9). Dunn *post hoc* tests with Bonferroni corrections revealed that African-Americans (Mdn = 3.0, IQR = 3.0) are significantly more likely to be the victim of hate-based harassment in general than Asian-Americans (Mdn = 1.0, IQR = 1.0)(*p*.adj = 0.032), White Americans (Mdn = 1.0, IQR = 1.0)(*p*.adj = 0.022), or Hispanic Americans (Mdn = 1.0, IQR = 1.0) (*p*.adj = 0.024). A chi-square test of independence found a significant relationship between a participant's ethnicity and the frequency of being a victim of ethnicity-based harassment (Table 10). From the standardized residuals, we find that this significant relationship is primarily due to differences in African and white American reported rates. African Americans (r = 2.70) are significantly more likely to be victimized by hate-based harassment due to ethnicity than expected based on overall proportions while white Americans (r = −4.33) are significantly more likely to be victimized by hate-based harassment due to ethnicity than expected based on overall proportions.

#### 5.2.2 Perpetration of hate-based harassment

**Figure 6:**
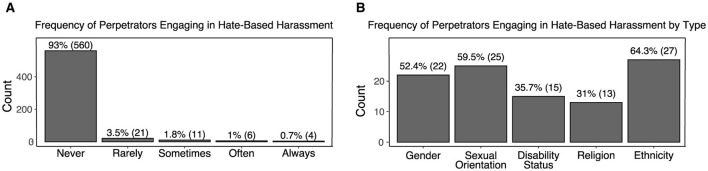
**(A)** Frequency of reported HBH perpetration in online games. **(B)** Proportion of players who report perpetrating HBH by type.

How often do players report engaging in hate-based harassment themselves? Of those surveyed, 7.0% report having harassed other players based on their victim's membership in a minority group (Figure 6). The median of the reported frequency of HBH perpetration across all types was 1.0 or *never* (IQR = 0.0) The most frequently reported type of hate-based harassment perpetrated by players was ethnicity-based (4.5%) followed by sexual orientation (4.2%), and gender (3.7%).

##### 5.2.2.1 Group differences

Kruskal Wallis tests were conducted to check for demographic group differences (age, gender, education, sexual orientation) and measures of association were calculated to test for relationships between HBH perpetration and personality traits (using Spearman's Rho) as well as gameplay motivations (using Gamma).

The test found significant effects of **age**, **gender**, and **education** on HBH perpetration (Table 11). However, Dunn *post hoc* tests with Bonferroni corrections show no significant differences among age and gender categories. For age, the largest group difference in medians is between individuals 18–21 years of age (Mdn = 1.0, IQR = 2.0) and individuals ages 22–25 years of age (Mdn = 1.0, IQR = 0.0), yet it does not rise to the level of significance (*p*.adj = 0.137). For gender, the largest group difference in medians is between males (Mdn = 1.0, IQR = 0.0) and females (Mdn = 1.0, IQR = 0.0), without reaching the level of significance (*p*.adj = 0.127). Regarding education, Dunn *post hoc* tests with Bonferroni alpha adjustments revealed that players with at least some graduate work are significantly more likely to perpetrate HBH than players who have only some high school or their GED (*p* = 0.003), players with only some college (*p* < 0.001), and players who have their bachelors degree (*p* =.001).

Table 11Kruskal-Wallis test results for effects of demographics on HBH perpetration.

**Table:** 

**Age**			**14–17 years old**		**18–21 years old**		**22–25 years old**	
H(2)	*p*.adj	ε^2^	Mdn	IQR	Mdn	IQR	Mdn	IQR
6.34	0.042	0.007	1.0_*a*_	0.0	1.0_*a*_	0.0	1.0_*a*_	0.0

**Table:** 

**Gender**			**Male**		**Female**		**Nonbinary**		**Prefer not to answer**	
H(3)	*p*.adj	ε^2^	Mdn	IQR	Mdn	IQR	Mdn	IQR	Mdn	IQR
8.19	0.042	0.009	1.0_*a*_	0.0	1.0_*a*_	0.0	1.0_*a*_	0.0	1.0_*a*_	0.0
**Education**			**Some high school/ GED**		**Some college**		**Bachelor's degree**		**Some graduate school/ graduate degree**	
H(3)	*p*.adj	ε^2^	Mdn	IQR	Mdn	IQR	Mdn	IQR	Mdn	IQR
17.36	<0.001	0.024	1.0_*a*_	0.0	1.0_*a*_	0.0	1.0_*a*_	0.0	1.0_*b*_	1.1
**Sexual orientation**			**Asexual**		**Bisexual**		**Heterosexual**		**Homosexual**	
H(3)	*p*.adj	ε^2^	Mdn	IQR	Mdn	IQR	Mdn	IQR	Mdn	IQR
3.79	0.285	0.001	1.0_*a*_	0.0	1.0_*a*_	0.0	1.0_*a*_	0.0	1.0_*a*_	0.0

Medians in a cell not sharing subscripts are significantly different from one another.

The Kruskal Wallis test found no significant effects of sexual orientation on HBH perpetration.

##### 5.2.2.2 Personality traits

Spearman's Rho was used to check for significant relationships between personality traits and HBH perpetration. Positive personality traits of emotional self-regulation (ρ = −0.14, *p*.adj = 0.004), communication (ρ = −0.21, *p*.adj < 0.001) and empathy (ρ = −0.24, *p*.adj < 0.001) were significantly negatively associated with HBH perpetration while negative personality traits of impulsivity (ρ = 0.17, *p*.adj < 0.001) and narcissism (ρ = 0.19, *p*.adj < 0.001) were significantly positively correlated with HBH perpetration.

##### 5.2.2.3 Gameplay motivations

Gamma was used to check for significant relationships between gameplay motivations (Yee, 2006) and HBH perpetration. Of the twelve motivations for online gameplay measured, only destruction was significantly associated with the perpetration of hate-based harassment (γ = 0.42, *p*.adj = 0.004).

### 5.3 RQ3. Can exposure to hate or particular gameplay habits lead to normalizing hate?

#### 5.3.1 Do online games normalize hate?

Our primary concern in this investigation is the normalization of hate in online games. In this section, we explore associations between online gameplay variables and normalization variables. The online gameplay variables we examine are: the amount of gameplay (hours/week) length of gameplay (in years), gamer identity, perceived expertise, and frequency of competition. The three operational definitions of normalizing hate used in this study are the following: diminished perceptions of dangerousness of hate speech (both general hate speech and specific types), perpetuating responses to it, and HBH perpetration. Together, these variables give us an overall sense of the relationships between online games and normalizing hate.

##### 5.3.1.1 Amount of gameplay

If online games normalize hate, then we might expect that how *frequently* a person games (in hours per week) may shape certain normalizing behaviors and attitudes.

###### 5.3.1.1.1 Perceived dangerousness of hate speech

We used Pearson Product Moment Correlation and Spearman's Rho (for ordinal variables) to examine the relationship between the amount of gameplay and perceptions of the dangerousness of hate speech (both generally and by type). We found no significant associations between the amount of gameplay and perceptions of the dangerousness of hate speech, generally or by type (Table 12).

**Table 12:** Associations between gaming habits and perceived dangerousness of hate speech.

**Generally**		**White supremacy**		**Antisemitism**		**Anti-Muslim**		**Anti-Asian**		**Misogyny**		**Racism**	
**Amount of gameplay**													
ρ	*p*.adj	ρ	*p*.adj	ρ	*p*.adj	ρ	*p*.adj	ρ	*p*.adj	r	*p*.adj	r	*p*.adj
−0.02	>0.999	−0.01	>0.999	−0.01	>0.999	−0.04	>0.999	−0.03	>0.999	−0.01	>0.999	−0.06	>0.999
**Length of gameplay**													
r	*p*.adj	ρ	*p*.adj	ρ	*p*.adj	ρ	*p*.adj	ρ	*p*.adj	r	*p*.adj	r	*p*.adj
0.06	>0.999	0.06	>0.999	0.05	>0.999	0.07	>0.999	0.02	>0.999	0.08	0.790	0.06	>0.999
**Gamer Identity**													
ρ	*p*.adj	γ	*p*.adj	γ	*p*.adj	γ	*p*.adj	γ	*p*.adj	γ	*p*.adj	γ	*p*.adj
0.01	>0.999	0.03	>0.999	0.02	>0.999	0.07	>0.999	0.02	>0.999	0.07	0.790	<0.01	>0.999
**Perceived expertise**													
r	*p*.adj	ρ	*p*.adj	ρ	*p*.adj	ρ	*p*.adj	ρ	*p*.adj	r	*p*.adj	r	*p*.adj
−0.03	>0.999	0.04	>0.999	−0.02	>0.999	−0.01	>0.999	−0.05	>0.999	−0.05	0.790	−0.02	>0.999
**Frequency of competition**													
ρ	*p*.adj	γ	*p*.adj	γ	*p*.adj	γ	*p*.adj	γ	*p*.adj	γ	*p*.adj	γ	*p*.adj
−0.04	>0.999	0.04	>0.999	−0.07	>0.999	−0.09	0.541	−0.09	0.524	−0.04	>0.999	−0.03	>0.999

Medians in a cell not sharing subscripts are significantly different from one another.

###### 5.3.1.1.2 Responses to hate speech

Spearman's Rho was used to assess the relationship between the amount of gameplay and responses to hate speech (by type and specific response). We found significant associations between the amount of gameplay and withdrawal responses as well as four specific response types (Table 14).

Heavier gaming is positively associated with *reporting the incident* (ρ = 0.13) but *negatively* associated with *withdrawal responses* both *generally* (ρ = -0.16) and specifically in terms of *leaving the match* (ρ = -0.13) *taking a break from the game*, (ρ = -0.15), and *quitting the game indefinitely* (ρ = -0.21).

###### 5.3.1.1.3 HBH perpetration

To examine the relationship between the amount of gameplay and HBH perpetration, we measured the strength of association between the amount of gameplay and HBH both general and by type. Using Spearman's Rho, we found no significant relationship between the amount of gameplay and HBH perpetration generally (ρ(600) = 0.11, *p*.adj = 0.099).

Simple logistic regressions between the amount of gameplay and HBH perpetration frequency by *type*, however, reveal significant associations between the amount of gameplay and both *disability*-based and *ethnicity*-based harassment (Table 13). Holding all other predictor variables constant, for every one-hour increase in the amount of gameplay per week, the odds of being a perpetrator of *disability-based harassment* increase by 5% and the odds of being a perpetrator of *ethnicity-based harassment* increase by 3%.

**Table 13:** Associations between gaming habits and HBH perpetration by type.

**Gaming habit**	**HBH type**	**B (SE)**	**Z**	***p*.adj**	**OR**	**95% CI**
Amount of gameplay	Gender	0.02(0.01)	1.81	0.844	1.02	[1.00, 1.05]
	Sexual orientation	0.03(0.01)	2.52	0.142	1.03	[1.00, 1.05]
	Disability status	0.05(0.01)	3.83	0.002	1.05	[1.02, 1.07]
	Religion	0.03(0.01)	2.38	0.208	1.03	[1.00, 1.06]
	Ethnicity	0.03(0.01)	2.90	0.044	1.03	[1.01, 1.05]
Length of gameplay	Gender	0.04(0.06)	0.66	>0.999	1.04	[0.93, 1.17]
	Sexual orientation	−0.01(0.06)	−0.14	>0.999	0.99	[0.89, 1.11]
	Disability status	0.17(0.07)	2.34	0.230	1.19	[1.03, 1.38]
	Religion	0.08(0.08)	0.99	>0.999	1.08	[0.93, 1.25]
	Ethnicity	0.02(0.05)	0.42	>0.999	1.02	[0.92, 1.14]
Gamer identity	Gender	−0.17(0.2)	−0.84	>0.999	0.84	[0.57, 1.28]
	Sexual orientation	0.05(0.21)	0.26	>0.999	1.06	[0.72, 1.63]
	Disability status	0.29(0.3)	0.98	>0.999	1.34	[0.78, 2.54]
	Religion	−0.24(0.26)	−0.93	>0.999	0.79	[0.49, 1.35]
	Ethnicity	0.14(0.21)	0.67	>0.999	1.15	[0.78, 1.77]
Perceived expertise	Gender	−0.33(0.24)	−1.38	>0.999	0.72	[0.45, 1.17]
	Sexual orientation	−0.11(0.23)	−0.49	>0.999	0.89	[0.57, 1.44]
	Disability status	−0.05(0.3)	−0.16	>0.999	0.95	[0.57, 1.78]
	Religion	−0.41(0.3)	−1.35	>0.999	0.66	[0.37, 1.23]
	Ethnicity	0.23(0.24)	0.94	>0.999	1.26	[0.79, 2.07]
Frequency of competition	Gender	0.37(0.2)	1.81	0.422	1.45	[0.98, 2.19]
	Sexual orientation	0.46(0.2)	2.35	0.114	1.58	[1.09, 2.36]
	Disability status	0.29(0.24)	1.22	>0.999	1.34	[0.84, 2.19]
	Religion	0.29(0.26)	1.12	>0.999	1.33	[0.81, 2.26]
	Ethnicity	0.4(0.19)	1.49	0.207	1.49	[1.04, 2.19]

Medians in a cell not sharing subscripts are significantly different from one another.

##### 5.3.1.2 Length of gameplay

Similarly, if online games normalize hate, then we might also expect that how long an individual has been gaming (in the number of years) may also shape certain normalizing behaviors and attitudes.

###### 5.3.1.2.1 Perceived dangerousness of hate speech

We used Pearson Product Moment Correlation and Spearman's Rho (for ordinal variables) to examine the relationship between length of gameplay (in years) and perceptions of the dangerousness of hate speech (both generally and by type). We found no significant associations between the length of gameplay and perceptions of the dangerousness of hate speech, generally or by type (Table 12).

###### 5.3.1.2.2 Responses to hate speech

Spearman's Rho was used to assess the relationship between the length of gameplay and responses to hate speech (by type and specific response). Similar to the findings for the amount of gameplay, we found significant associations between the length of gameplay and withdrawal responses as well as four specific response types (Table 14).

**Table 14:** Responses to hate speech by gaming habits.

	**Amount of gameplay**		**Length of gameplay**		**Gamer identity**		**Perceived expertise**		**Competition**	
**Grouped responses**	ρ	***p*** **.adj**	ρ	***p*** **.adj**	ρ	***p*** **.adj**	*r*	***p*** **.adj**	ρ	***p*** **.adj**
Productive responses	0.07	>0.999	0.07	>0.999	0.09	0.772	0.05	>0.999	0.10	0.365
Withdrawal responses	−0.16	0.002	−0.17	0.001	−0.15	<0.001	−0.18	<0.001	−0.23	<0.001
Perpetuating responses	0.12	0.180	0.04	>0.999	0.06	>0.999	0.12	0.137	0.20	<0.001
**Responses**	ρ	***p*** **.adj**	ρ	***p*** **.adj**	γ	***p*** **.adj**	ρ	***p*** **.adj**	γ	***p*** **.adj**
Refocus chat on game	0.015	>0.999	0.013	>0.999	0.06	>0.999	0.05	>0.999	0.08	>0.999
Support victim	−0.03	>0.999	−0.081	>0.999	−0.04	>0.999	0.13	>0.999	−0.01	>0.999
Report	0.13	<0.001	0.15	0.010	0.16	0.011	0.02	>0.999	0.08	>0.999
Call out speaker	0.04	>0.999	0.05	>0.999	0.042	>0.999	0.03	>0.999	0.08	0.006
Ignore it	0.09	0.823	0.07	>0.999	0.11	>0.782	0.05	>0.782	−0.05	>0.999
Withdrawal from chat	−0.06	>0.999	−0.10	0.538	−0.01	>0.999	−0.06	>0.999	−0.17	0.002
Mute the speaker	−0.06	>0.999	−0.09	0.893	−0.01	>0.999	−0.08	>0.999	−0.13	0.052
Leave match	−0.24	<0.001	−0.14	0.024	−0.29	<0.001	−0.14	<0.028	−0.22	<0.001
Take break from game	−0.15	<0.001	−0.17	0.001	−0.20	0.001	−0.16	0.002	−0.28	<0.001
Quit game indefinitely	−0.21	<0.011	−0.18	<0.001	−0.30	<0.001	−0.17	0.001	−0.20	>0.006
Laughing	0.10	0.516	0.05	>0.999	0.11	>0.874	0.14	0.022	0.15	>0.016
Toxicity in return	0.13	0.075	0.044	>0.999	0.09	>0.999	0.12	0.122	−0.02	<0.001
Join in	0.06	>0.999	−0.017	>0.999	−0.04	>0.999	0.01	>0.999	0.10	>0.999
Share with others who might agree	−0.04	>0.999	−0.06	>0.999	−0.13	0.917	−0.07	>0.999	0.10	>0.999
Other	0.06	>0.999	0.09	>0.999	0.21	>0.999	0.19	0.730	0.20	0.680

Means/medians in a cell not sharing subscripts are significantly different from one another.

Longer term gameplay is *positively* associated with *reporting the incident* (ρ = 0.15) but *negatively* associated with *withdrawal responses* both *generally* (ρ = −0.17) and specifically in terms of *leaving the match* (ρ = −0.14) *taking a break from the game*, (ρ = −0.17), and *quitting the game indefinitely* (ρ = −0.18).

###### 5.3.1.2.3 HBH perpetration

To examine the relationship between length of gameplay and HBH perpetration, we measured the strength of association between length of gameplay and HBH both general and by type. Using Spearman's Rho, we found no significant relationship between the length of gameplay and HBH perpetration generally (ρ(600) =.004, *p*.adj>0.999). Simple logistic regressions between the amount of gameplay and HBH perpetration frequency by type also revealed no significant associations in terms of the adjusted p-values although the confidence interval for disability-based harassment perpetration does not contain 1.0, suggesting that the true population odds ratio is above 1.0. Thus it is indeed possible that length of gameplay may increase the odds ratio of an individual engaging in disability-based harassment by 19% but the adjusted p-value is too low to detect the difference statistically (Table 13).

##### 5.3.1.3 Gamer Identity

What defines a “gamer” is largely vested in one's own perception of the term, but is often framed around characteristics like gender, sexuality, and race, as well as the stigmas surrounding games and gaming culture (Shaw, 2012). However, the stereotypical gamer is commonly portrayed as predominantly young, white, heterosexual, and male. Events like gamergate have brought attention to a vocal subset of players who strongly identify with this depiction of the term (Paaßen et al., 2017) and perceive the diversification of gaming culture as an attack on their status quo (Aghazadeh et al., 2018). Prior literature suggests that players fusing this type of gamer identity with their own may help normalize these problematic behaviors and attitudes in the spaces they inhabit (Kowert et al., 2022). Here, we examine both gamer identity and the more neutral construct of perceived expertise to investigate potential relationships among feelings and perceptions of belonging and recognition within the broader game community and the potential normalization of hate through online games.

###### 5.3.1.3.1 Perceived dangerousness of hate speech

We used Spearman's Rho and Gamma (for ordinal variables) to examine the relationship between gamer identity and perceptions of the dangerousness of hate speech (both generally and by type). We found no significant associations (Table 12).

###### 5.3.1.3.2 Responses to hate speech

Spearman's Rho and Gamma (for ordinal variables) were used to assess the relationship between gamer identity and responses to hate speech (by type and specific response). Again, we found significant associations between gamer identity and withdrawal responses as well as four specific response types (Table 14).

Gamer identity is positively associated with *reporting the incident* (γ = 0.16) but *negatively* associated with *withdrawal responses* both generally (ρ = −0.15) and specifically in terms of leaving the match (γ = −0.29) *taking a break from the game*, (γ = −0.20), and *quitting the game indefinitely* (γ = −0.30).

###### 5.3.1.3.3 HBH perpetration

To examine the relationship between gamer identity and HBH perpetration, we measured the strength of association between gamer identity and HBH both general and by type. Using Gamma, we found no significant relationship between the gamer identity and HBH perpetration generally (γ = −0.04, *p*.adj>0.999). Simple logistic regressions between gamer identity and HBH perpetration frequency by type also revealed no significant associations in terms of the adjusted *p*-values (or, in this case, confidence intervals) (Table 13).

##### 5.3.1.4 Perceived expertise

Next, we examine *perceived expertise* as a potentially more neutral formulation than *gamer identity* of the perception of belonging and recognition within the game community.

###### 5.3.1.4.1 Perceived dangerousness of hate speech

Pearson Product Moment Correlations and Spearman's Rho (for ordinal variables) were used to examine the relationship between perceived expertise and perceptions of the dangerousness of hate speech (both generally and by type). We found no significant associations (Table 12).

###### 5.3.1.4.2 Responses to hate speech

Pearson Product Moment Correlation and Spearman's Rho (for ordinal variables) were used to assess the relationship between perceived expertise and responses to hate speech (by type and specific response). We found significant associations between perceived expertise and withdrawal responses generally as well as four specific response types (Table 14). Perceived expertise is positively associated with *laughing* (ρ = 0.14) in response to hate speech but *negatively* associated with *withdrawal responses* both generally (r = −0.18) and specifically in terms of *withdrawing from in-game chat* (ρ = −0.14), *leaving the match* (ρ = −0.14), *taking a break from the game*, (ρ = −0.16), and *quitting the game indefinitely* (ρ = −0.17).

###### 5.3.1.4.3 HBH perpetration

Using Spearman's Rho, we examined the relationship between perceived expertise and HBH perpetration generally and found no significant relationship between the length of gameplay and HBH perpetration generally (ρ(600) = 0.01, *p*.adj>0.999). We then conducted simple logistic regressions between perceived expertise and HBH perpetration frequency by type (Table 13). We found no significant associations.

##### 5.3.1.5 Frequency of competition

Next, we examine potential associations between the frequency with which a player engages in *competition* in online games and our three variables representing the normalization of hate. Prior findings describe patterns of racism, sexism, and homophobia found in ranked play and esports (Costa et al., 2023; Ruotsalainen and Friman, 2018; Sengün et al., 2019), with young players expressing an acceptance of this conduct as simply being part of the environment.

###### 5.3.1.5.1 Perceived dangerousness of hate speech

We used Spearman's Rho and Gamma (for ordinal variables) to examine the relationship between frequency of competition and perceptions of the dangerousness of hate speech (both generally and by type). We found no significant associations (Table 12).

###### 5.3.1.5.2 Responses to hate speech

Spearman's Rho and Gamma (for ordinal variables) were used to assess the relationship between frequency of competition and responses to hate speech (by type and specific response), revealing significant associations between frequency of competition and responses to hate speech both generally and by type (Table 14). Frequency of competition is positively associated with *perpetuating responses generally* (ρ = 0.20) as well as the specific perpetuating responses of *laughing* (γ = 0.15) and *being toxic in return* (γ = 0.22), but it is also *positively* associated with the more productive response of *calling out speaker* (γ = 0.08). Competition frequency is *negatively* associated with *withdrawal responses* both *generally* (ρ = −0.23) and specifically in terms of *withdrawing from in-game chat* (γ = −0.17), *leaving the match* (γ = −0.22), *taking a break from the game*, (γ = −0.28), and *quitting the game indefinitely* (γ = −0.20).

###### 5.3.1.5.3 HBH Perpetration

We calculated Gamma to measure the relationship between the frequency of competition and HBH perpetration generally and found a significant positive relationship with HBH perpetration generally (γ = 0.32, *p*.adj = 0.025). Individuals who engage more frequently in competitive matches are significantly more likely to harass other players who are marginalized because of their identity. Simple logistic regressions between frequency of competition and HBH perpetration frequency by type reveal no additional significant associations based on adjusted p-values although the confidence intervals for HBH perpetration based on sexual orientation and on ethnicity do not contain 1.0, suggesting that the true population odds ratio is above 1.0. It is possible, then, that the frequency of competition may indeed increase the odds ratio of an individual perpetrating sexual orientation-based harassment by 58% and ethnicity-based harassment by 49% but the adjusted *p*-value is too low to detect the difference statistically (Table 13).

#### 5.3.2 Does exposure to hate normalize hate?

Now that we have examined the potential role of gameplay habits in normalizing hate in online games, we turn toward our final set of tests, exploring the potential consequences of exposure to hate speech and hate-based harassment that online games currently enable. What are the consequences of exposure to in-game hate speech and hate-based harassment, particularly for teens and young adults? In this section, we examine associations between exposure to hate speech, exposure to HBH as a bystander, and HBH victimization and our three normalization variables examined in the previous section.

##### 5.3.2.1 Exposure to hate speech

Earlier in this paper, we reported that more than four-fifths (84.9%) of adolescent participants in this study reported encountering some form of hate speech while playing online. Here, we investigate whether this exposure itself has potentially negative consequences.

###### 5.3.2.1.1 Perceived dangerousness of hate speech

Does exposure to hate speech lead to diminished perceptions of its dangerousness? To examine this question, we used Spearman's Rho and Gamma (for ordinal variables) to examine the relationship between exposure to hate speech and perceptions of the dangerousness of hate speech (both generally and by type) (Table 15).

**Table 15:** Associations between exposure to hate speech and perceptions of the dangerousness of hate speech (generally and by type).

**Perceptions of the dangerousness of hate speech**	**r**	***p*.adj**
Generally	0.05	>0.999
Misogyny	0.10	0.155
Racism	0.06	>0.999
	γ	***p*** **.adj**
White supremacy	0.06	>0.999
Anti-Semitism	−0.01	>0.999
Anti-Muslim	−0.02	>0.999
Anti-Asian	0.16	0.10

Exposure to hate speech generally is not significantly associated with perceptions of its overall dangerousness. Among the relationships examined between exposure to specific types of hate speech and perceptions of the dangerousness of hate speech of those same types, only one reaches the point of significance: *anti-Asianism*. Exposure to anti-Asian hate speech is *positively* associated with perceptions of its dangerousness (γ = 0.16). Rather than diminishing perception of its dangers, exposure to anti-Asian hate speech increases it.

###### 5.3.2.1.2 Responses to Hate Speech

Spearman's Rho was calculated to assess the relationship between exposure to hate speech and responses to it (both type and specific responses) (Table 16).

**Table 16:** Associations between exposure to hate speech and responses to hate speech (by type and specific responses).

**Responses to hate speech**	**ρ**	***p*.adj**
Productive responses	0.24	<0.001
Withdrawal responses	0.11	0.306
Perpetuating responses	0.18	<0.001
Refocus chat on game	0.10	0.779
Support victim	0.20	<0.001
Report	0.10	>0.999
Call out speaker	0.21	<0.001
Ignore it	−0.06	>0.999
Withdrawal from chat	0.05	>0.999
Mute the speaker	0.01	>0.999
Leave match	0.18	<0.001
Take break from game	0.15	0.016
Quit game indefinitely	0.21	<0.001
Laughing	0.03	>0.999
Toxicity in return	0.22	<0.001
Join in	0.17	0.002
Share with others who might agree	0.17	<0.001
Other	0.11	>0.999

Exposure to hate speech is *positively* associated with both *productive responses* to hate speech generally (ρ = 0.24) and by the specific response, including: *supporting the victim* (ρ = 0.20) and *calling out the speaker* (ρ = 0.21). It is also *positively* associated with both *perpetuating responses* to hate speech generally (ρ = 0.18) and by specific response, including: *being toxic in return* (ρ = 0.22), *joining in*(ρ = 0.17), and *sharing what was said with others who might agree with it* (ρ = 0.17). While such exposure is not significantly associated with withdrawal responses generally, it is positively associated with specific forms of withdrawal responses, namely: *leaving the match* (ρ = 0.18), *taking a break from the game* (ρ = 0.15), and even *quitting the game indefinitely* (ρ = 0.21).

###### 5.3.2.1.3 HBH perpetration

Spearman's Rho was used to measure the relationship between exposure to hate speech and HBH perpetration generally. Increased exposure to hate speech is significantly associated with increased perpetration of hate-based harassment regardless of type (ρ(600) = 0.13, *p* = 0.013). Simple logistic regressions were conducted to examine the associations between exposure to types of hate speech and HBH perpetration frequency of the same type (Table 17). For every one point of exposure to white supremacy hate speech (on a 5-point ordinal Likert scale), the odds of a player engaging in hate-based harassment based on religion go up by 128%. Based on the confidence interval rather than the adjusted p-value for the simple logistic regression between white supremacy and ethnicity-based HBH, it may also be the case that for every one point of exposure to white supremacy hate speech, the odds of a player engaging in hate-based harassment based on ethnicity also go up by 52%. For every one point of exposure to antisemitic hate speech, the odds of a player engaging in hate-based harassment based on religion go up by 96%. Finally, for every one point of exposure to anti-Muslim hate speech, the odds of a player engaging in hate-based harassment based on religion go up by 92%.

**Table 17:** Associations between exposure to hate speech and HBH perpetration by type.

**Hate speech type**	**HBH type**	**B (SE)**	**Z**	***p*.adj**	**OR**	**95% CI**
White supremacy	Ethnicity	0.42(0.18)	2.39	0.167	1.52	[1.06, 2.12]
White supremacy	Religion	0.82(0.22)	3.73	0.002	2.28	[1.46, 3.53]
Anti-semitism	Religion	0.67(0.22)	3.06	0.022	1.96	[1.25, 3.00]
Anti-Muslim	Religion	0.65(0.21)	3.06	0.022	1.92	[1.26, 2.95]
Anti-Asian	Ethnicity	0.28(0.15)	1.88	0.602	1.33	[0.99, 1.78]
Misogyny	Gender	0.15(0.18)	0.80	>0.999	1.16	[0.79, 1.65]
Racism	Ethnicity	0.36(0.19)	1.89	0.584	1.43	[0.96, 2.03]

##### 5.3.2.2 Bystander exposure to hate-based harassment

More than four-fifths (82.2%) of adolescent players are bystanders to hate-based harassment in online games. What are the impacts? Here, we investigate the potential role of such experiences in normalizing hate.

###### 5.3.2.2.1 Perceived dangerousness of hate speech

Spearman's Rho was used to assess the association between bystander HBH exposure and the perceived dangerousness of hate speech overall. There is a significant positive association between bystander HBH exposure and the perceived dangerousness of hate speech regardless of type (ρ(600) = 0.15, p = 0.002).

###### 5.3.2.2.2 Responses to hate speech

Spearman's Rho and Gamma (for ordinal variables) were used to assess the relationship between bystander HBH exposure and responses to hate speech (both type and specific responses) (Table 18). Exposure to hate-based harassment as a bystander is positively associated with productive responses to hate speech both generally (ρ = 0.23) and by specific response, including: *reporting it* (ρ = 0.20) and *calling out the speaker* (ρ = 0.24). It is also associated with a specific perpetuating response: *being toxic in return* (ρ = 0.23).

**Table 18:** Associations between bystander HBH exposure and responses to hate speech (by type and specific responses).

**Responses to hate speech**	**ρ**	***p*.adj**
Productive responses	0.23	<0.001
Withdrawal responses	−0.05	>0.999
Perpetuating responses	0.11	0.410
	γ	***p*** **.adj**
Refocus chat on game	0.02	>0.999
Support victim	0.12	0.858
Report	0.20	<0.001
Call out speaker	0.24	<0.001
Ignore it	−0.12	0.277
Withdrawal from chat	−0.02	>0.999
Mute the speaker	−0.05	>0.999
Leave match	0.07	>0.999
Take break from game	−0.02	>0.999
Quit game indefinitely	0.09	>0.999
Laughing	−0.04	>0.999
Toxicity in return	0.23	<0.001
Join in	0.04	>0.999
Share with others who might agree	0.15	0.508
Other	0.12	>0.999

###### 5.3.2.2.3 HBH perpetration

Gamma was used to assess the association between bystander HBH exposure and the HBH perpetration. There is a significant positive association between the two (γ =.37, p =.011).

##### 5.3.2.3 Victim exposure to hate-based harassment

Finally, we examine the potential relationships between victim HBH exposure and normalizing hate. With 38.2% of participants reporting having been personally targeted for hate-based harassment, the potential role of such victimization in the dynamics of normalizing hate speech and harassment warrants examination.

###### 5.3.2.3.1 Perceived dangerousness of hate speech

Spearman's Rho was used to assess the association between victim HBH exposure and the perceived dangerousness of hate speech overall. No significant association was found (ρ(600) = 0.09, *p* = 0.296).

###### 5.3.2.3.2 Responses to hate speech

Spearman's Rho and Gamma (for ordinal variables) were used to assess the relationship between victim HBH exposure and responses to hate speech (both type and specific responses) (Table 19). Exposure to hate-based harassment as a victim is *positively* associated with productive responses to hate speech both generally (ρ = 0.27) and by the specific response, including *asking for support* (ρ = 0.33), *reporting it* (ρ = 0.20) and *calling out the speaker* (ρ = 0.32). Exposure as a victim is also associated with specific perpetuating responses to hate speech both generally (ρ = 0.15) and by the specific response: *being toxic in return* (ρ = 0.28). Such victimization is also associated with the most extreme withdrawal response we surveyed: *quitting the indefinitely* (ρ = 0.24).

**Table 19:** Associations between victim HBH exposure and responses to hate speech (by type and specific responses).

**Responses to hate speech**	**ρ**	***p*.adj**
Productive responses	0.27	<0.001
Withdrawal responses	−0.03	>0.999
Perpetuating responses	0.15	0.019
	γ	***p*** **.adj**
Refocus chat on game	0.05	>0.999
Ask for support	0.33	<0.001
Report	0.20	0.004
Call out speaker	0.32	<0.001
Ignore it	−0.15	>0.999
Withdrawal from chat	−0.08	>0.999
Mute the speaker	−0.11	>0.999
Leave match	0.06	>0.999
Take break from game	0.13	0.939
Quit game indefinitely	0.24	0.031
Laughing	−0.03	>0.999
Toxicity in return	0.28	<0.001
Join In	0.15	>0.999
Share with others who might agree	0.20	0.136
Other	0.01	>0.999

###### 5.3.2.3.3 HBH perpetration

Gamma was used to assess the association between victim HBH exposure and HBH perpetration. There is a significant positive association between the two (γ = 0.62, *p* < 0.001).

## 6 Discussion

The goal of this survey study was to assess the prevalence of hate speech and hate-based harassment on online game platforms, adolescent player perceptions and responses to the problem, and whether such encounters might contribute to a normalization of hateful rhetoric and ideologies among gaming youth. The results show that, while hate speech encounters are rare, the majority of players are exposed at some point during online gameplay. How dangerous participants perceived such rhetoric to be varied greatly based on personal factors; for instance (and potentially as a reflection of the “straight white young male gamer” stereotype), male and heterosexual players found hate speech significantly less dangerous than did players of other genders and sexual orientations.

In response to hate speech, participants generally chose to either act productively - reporting and calling out the inflammatory event, supporting the victim, or refocusing the group on the match at hand - or to withdraw themselves from the interaction completely. Again, there were specific group differences. Teenage players laughed off such events more often than adult players, possibly hinting at a generational difference between those growing up at a time when toxic online gamer culture had already been established and those who did not. Male players were more likely to respond in a perpetuating manner, while female players were more likely to withdraw. This aligns with the existing literature on gender in games that documents the lengths women have gone to in order to hide their gender identity to avoid misogynistic comments (Madden et al., 2021; Fox and Tang, 2017). Education level did not have a significant relationship to players' perceptions or responses to hate speech.

The vast majority of adolescent players had experienced being a bystander to hate-based harassment, particularly in regards to gender. Over a third also reported being a victim, with ethnicity being the most common basis of harassment. The distribution of victimization among demographic groups is fairly unsurprising: female players are significantly more likely to be harassed for their gender than male players; asexual, bisexual, and homosexual players are more likely to be harassed for their sexual orientation than heterosexuals; and players with disabilities are more likely to be harassed for their disability status than those without. Muslims are more likely to be harassed than expected and atheists and agnostics were less likely. African American/Black players were more likely to be harassed for their ethnicity than all other ethnicities and are at notably more risk of facing race-based harassment than white players are.

Compared to witnessing or directly experiencing hate-based harassment, a very small percentage of players admit to harassing others for their identity. Participants with experience in graduate-level education were reported more likely to be perpetrators of hate-based harassment than those of lower education levels. Players with positive personality traits like emotional self-regulation, communication, and empathy are less likely to be perpetrators of hate-based harassment, while those with negative traits like impulsivity and narcissism are more likely. Of the motivations provided, only one, destruction, was associated with perpetrating hate-based harassment.

Players who identify more strongly as a “gamer”, who logged more hours per week, and who have played for a longer period of time overall were more likely to report than to withdraw from hateful conduct. Those who perceive themselves to be experts laugh in response to hate speech more often and withdraw less. Competitive gamers reported responding both productively and in a perpetuating way.

Exposure to hate speech correlated to increased perceptions of dangerousness primarily in regard to anti-Asian rhetoric, although this study's predominantly Asian sample may have skewed this result. Exposure correlates to increases in both productive and perpetuating responses. It also correlates with the increased perpetration of hate-based harassment. Witnessing hate speech positively correlates with an increased perception of dangerousness, while both witnessing and being a victim of hate speech were associated with an increase in productive responses.

These results paint a current portrait of hate speech and hate-based harassment in online games consistent with the emerging literature. White, male, and heterosexual players are less targeted by hateful rhetoric for their identity characteristics than players from marginalized groups, and so are understandably less likely to feel threatened by, confront, or shy away from such rhetoric when they encounter it. Players with higher levels of impulsivity and narcissism, or those who are motivated by destruction or frequently engage in competitive brackets, are more likely to engage in hateful conduct than those with more positive personality traits and broader motivations for play. Such findings punctuate the need for greater awareness among game players and greater diversity among game designers, signaling the blind spots of game creators and consumers, the majority of whom continue to be mostly white, cisgender, and male (Bezio, 2018; Maloney et al., 2019; Ratan et al., 2015).

While instances of exposure to hateful rhetoric are rare, the accumulation of such exposures over a prolonged period of time seems to correlate to complex long-term impacts. Veteran players appear more willing to report instances of hate but are less likely to limit their play in response, suggesting that exposure is not enough to turn them off from playing the game. By contrast, newer or more marginalized players are more likely to withdraw and even drop a game title entirely when hate speech goes unchecked.

These factors coincide with the finding that more frequent exposure to hate speech seems to correlate with increased perpetration of hate-based harassment. White supremacist speech in particular has a dramatic association with greater odds of engaging in race-based and religion-based harassment, increasing the odds of perpetration by 52.1% and 127.7% respectively. Such large numbers are difficult to ignore. Antisemitic and anti-muslim hate speech also correlate to dramatic increases in religion-based harassment, increasing the odds of perpetration by similarly large numbers (95.6% and 92.4% respectively). Taken together, these consistent findings at a more granular level of the analysis suggest that exposure to hate might correspond to greater levels of hate overall.

However, it must be noted that exposure to hate in the form of specific types of hate speech (anti-Asian and misogynistic), witnessing the hate-based harassment of others, or being the target oneself are all associated with increased perception of its dangerousness, raising the alarm among individuals as to the dangers of speech and behavior that demean individuals on the basis of inherent characteristics of who they are, be that ethnicity or gender identity or similar grounds. And players who more frequently witness in-game hate-based harassment of others or are targeted by hate-based harassment themselves are more likely to also engage in productive responses, potentially signaling to those involved in the act that such speech and actions are unacceptable in the game space and community.

Thus, this study's findings suggest that if hate has truly been normalized within online gaming spaces, its presence is seemingly maintained by members who are less impacted by hate being the inhabitants who return most often. Those who are exposed to hateful conduct seem to withdraw from play while those who stay are more likely to report but less likely to withdraw. Members of minority groups are targeted more often, are more likely to withdraw in response, and perceive greater danger from hateful acts; this aligns with the finding that frequently-targeted participants (or those who witness fellow group members being victimized) seem to have an increased perception of danger as well. While further work is needed to suggest any causal trends regarding shifts in hateful conduct occurring in these spaces, these findings do support the assertion that targeted individuals remove themselves from these environments more often, allowing those less affected by hateful conduct to define and maintain the culture within the community. We argue that this maintenance acts as the continued normalization of hate. Future research should investigate trends among who stays and who leaves online game communities over the long term, their reasons for doing so, and the repercussions of leaving hate speech and hate-based harassment to persist unabated.

Surveys such as these have significant limitations. While we sought to make our demographic items as inclusive as possible, unfortunate omissions on our part (for instance, allowing respondents to specify between transgender and cisgender) left out important context for several factors. The majority of respondents were recruited from the host university, with a local population containing a higher proportion of individuals from minoritized groups with positive personality traits. Thus, the results of this study might not be generalizable to other populations. Additionally, given the nature of the items, the data are self-reported and not direct observations, limiting their reliability. Moreover, because they gather cross-sectional data at a single moment in time, they cannot test causal relationships between two variables but only associations, a necessary but insufficient prerequisite for causal claims on their own. In this light, perhaps the most important contribution of this investigation is to direct our attention to those relationships that warrant more in-depth investigation via experimental work that might substantiate the suggestive relationships found.

## Data Availability

The raw data supporting the conclusions of this article will be made available by the authors, without undue reservation.
